# Interstitial lung disease in children – genetic background and associated phenotypes

**DOI:** 10.1186/1465-9921-6-32

**Published:** 2005-04-08

**Authors:** Dominik Hartl, Matthias Griese

**Affiliations:** 1 Pediatric Pneumology, Childrens' hospital of the Ludwig-Maximilians-University, Munich, Germany

**Keywords:** interstitial lung disease, children, surfactant-protein C, ABCA3, mutations

## Abstract

Interstitial lung disease in children represents a group of rare chronic respiratory disorders. There is growing evidence that mutations in the surfactant protein C gene play a role in the pathogenesis of certain forms of pediatric interstitial lung disease. Recently, mutations in the ABCA3 transporter were found as an underlying cause of fatal respiratory failure in neonates without surfactant protein B deficiency. Especially in familiar cases or in children of consanguineous parents, genetic diagnosis provides an useful tool to identify the underlying etiology of interstitial lung disease. The aim of this review is to summarize and to describe in detail the clinical features of hereditary interstitial lung disease in children. The knowledge of gene variants and associated phenotypes is crucial to identify relevant patients in clinical practice.

## Review

### Interstitial lung disease in children

Interstitial lung disease (ILD) in children represents a heterogeneous group of rare respiratory disorders characterised by restrictive lung disease and diffuse pulmonary infiltrates. Affected children usually present with dry cough, dyspnoea and tachypnoea. Physical findings show elevated resting respiratory rate and rales (or crackles) mostly more pronounced in the basal segments of the lungs. In children suffering from severe ILD, chronic hypoxemia, cyanosis, finger clubbing and growth failure occur [1,2]. High-resolution computed tomography provides detailed diagnostic information about the extent and distribution of the lung pathology and has become the most relevant imaging technique for ILD in children [3]. However, the diagnostic gold standard is still the lung biopsy where the characteristic histological findings of inflammation in the pulmonary interstitium with wall thickening by inflammatory cells and/or fibrosis can be found. At present, no pathognomonic laboratory criteria for the diagnosis of ILD in children are available [4]. ILD is associated with pulmonary inflammation which can resolve completely, partially or can progress to fibrosis. The factors determining the progression of ILD are not well understood but an interaction between genetic background and environmental modifiers is suggested [1,5-7].

In children, ILD is less frequent and comprises a broader spectrum of disorders with a more variable clinical course compared to adults. In addition, there are special disease entities which predominantly occur in children, like the recently described pulmonary interstitial glycogenosis [8], the neuro-endocrine cell hyperplasia of infancy [9] and the genetic disorders of surfactant metabolism [10]. Most of the information on ILD in childhood is based on studies performed in adults. In the recent years, however, there is increasing evidence that ILD in children differs substantially from adult ILD [2]. PediatricILD occur in the context of lung development and are most frequent diagnosed in the first year of life when alveolar development is taking place [11,12]. The combination of epidemiological rarity, clinical heterogenity and genetic background makes the classification of ILD in children difficult. Thus, studies up to now included only limited numbersof patients, except of one recent study encompassing 185 children [13], and no randomised controlled trials have been performed to evaluate treatment strategies.

Mutations in surfactant protein B (SP-B) cause fatal respiratory distress in neonates [14]. Recently, mutations in the SP-C gene and in the ATP-binding cassette protein A3 (ABCA3) gene were found to be associated with pediatric ILD [15-17]. SP-C gene variants presented as a variable clinical phenotype. While children with SP-C mutations showed mostly non-specific interstitial pneumonitis (NSIP) except of two cases with combined pulmonary alveolar proteinosis (PAP), adults with SP-C mutations suffered from usual interstitial pneumonitis (UIP) or desquamative interstitial pneumonitis (DIP) or remained asymptomatic. ABCA3 mutations were associated with fatal respiratory failure in neonates without SP-B or SP-C deficiency and with non-fatal ILD in one older child. These infants showed a diagnosis of PAP, DIP or chronic pneumonitis of infancy (CPI) [18].

The aim of this review is to summarize and describe in detail the reported clinical courses of hereditary ILD in children. At first, the role of **surfactant protein B **in ILD is described. It is delineated how SP-B deficiency results in lung disease in mouse models and in humans. Reported single cases with partial SP-B deficiency are discussed in detail. Similarly, the role of **surfactant protein C **in ILD is reviewed and a model of surfactant protein C associated lung disease is described. Finally, the roles of **ABCA3 **and of further genes involved in surfactant protein expression, lung morphogenesis and lamellar body synthesis are discussed. The diversity of gene variants and associated phenotypes illustratesthe overlap between genetic defect, environmental influence and the resulting respiratory disease. Knowledge on the clinical features of hereditary ILD is crucial to identify relevant patients for genetic analysis in clinical practice.

#### Surfactant

The alveolus of the human lung tends to collapse at the end of the expiration due to high surface tension. A phospholipid film, named surfactant, holds alveolar stability at the air-liquid interface by reducing surface tension. Surfactant is a mixture of lipids and proteins, which contains 70 – 80% phospholipids, 10% protein and about 10% of neutral lipids. When surfactant is lacking at birth, the alveoli collapse, the alveolo-capillary membranes become leaky and intra-alveolar hyaline membranes occur, termed as respiratory distress syndrome (RDS). Surfactant is synthesized and secreted by type II epithelialcells. Surfactant proteins can be divided into the hydrophobic proteins SP-B and SP-C and the hydrophilic proteins SP-A and SP-D. While SP-A and SP-D play essential roles in the pulmonary immune defense, SP-B and SP-C lower the surface-tension and facilitate formation and stabilization of the surfactant film [19,20].

### Surfactant protein B

#### Structure and biosynthesis

Human SP-B is encoded on the short arm of chromosome 2 and spans about 10 kilobases. The SP-B gene contains 11 exons, with the 11th exon being untranslated, and is transcribed into a ~2000 base pair mRNA [21-23]. Corticosteriods were found to enhance the SP-B transcription [24], while Transforming Growth Factor-β (TGF-β), NO and insulin inhibited the transcriptional process [25,26]. The SP-B gene is expressed in type II cells and in non-ciliated bronchiolar epithelial cells, but only type II cells are able to fully process the inital translation product, pro-SP-B, to functionally active, mature SP-B. In a first step, the SP-B mRNA is translated into a preproprotein of 381-amino acids [23,27-29]. After removal of the first 23 amino acids, the remaining proprotein (proSP-B) is proteolytically processed at the amino-, and carboxy-terminal ends to the mature SP-B of 79 amino acid. The cysteine protease, cathepsin H and the aspartyl protease, Napsin A, highly expressed in type II epithelial cells, have been implicated recently in processing of the SP-B and SP-C propeptides [30-32].

#### Surfactant protein B deleted mice

After birth, SP-B deleted mice showed normal respiratory efforts but failed to inflate the lungs, were cyanotic and died rapidly due to severe respiratory failure [33]. Histologically, their lungs showed a normal morphogenesis without signs of inflammation. Pulmonary function testing revealed decreased lung volumesand an absence of residual volume at end-expiration, similar to neonates with RDS. Furthermore, no proSP-B or SP-B protein were detected and no tubular myelin and lamellar bodies were found in lungs of these mice. Interestingly, an aberrant form of proSP-C of 12-kd and abundance of SP-A and SP-C mRNA were detected in these lungs, but fully processed SP-C protein was found to be decreased. Type II alveolar cells of these mice contained notypical lamellar bodies and showed enlarged multivesicularbodies with small lipid vesicles, which suggests a role for SP-B in the packing of surfactantphospholipids into lamellar bodies [34,35]. These findings in SP-B deficient mice indicate thatSP-B is critical for reduction of surface tension in the alveolus, for the proteolytic processing of proSP-C, for the arrangementof surfactant phospholipids in lamellar bodies and for lung functionduring the early neonatal period.

#### Surfactant protein B deficiency in human lung disease

SP-B deficiency was the first reported genetic cause of lethal RDS in infants [36]. A full-term newborn died in the neonatal period from unclear lung disease. An open lung biopsy revealed the histopathological features of congenital PAP, a pathology which had been observed previously in several full-term neonates with severe respiratory failure, mostly with a family history of neonatal respiratory disease [37], suggesting an autosomal-recessive inheritance. In lung tissue of the patient, mature SP-B was undetectable whereas SP-A and proSP-C were regularly found. Genetic analysis revealed that the affected infant was homozygous for a frameshift mutation in codon 121 (termed 121ins2 mutation) in the SP-B gene [38,39]. The 121ins2 loss-of-function mutation is suggested to account for up to two-thirds of the mutant alleles identified in the SP-B locus. At least 27 loss of function mutations were found in the SP-B gene that resulted in neonatal RDS [40-48]. Since most of these mutations were unique to the index families the sensitivity of genetic counseling is limited. The incidence of hereditary SP-B deficiency is not known, but an allele frequency of 1 per 1000–3000 individuals for the 121ins2 mutation is suggested [45,47-49]. The phenotype of infants with hereditary SP-B deficiency is described as typically full-term neonates with severe RDS who are refractory to standard therapies, includingventilation and surfactant replacement. Chest X-rays usually show diffuse ground glass pattern in both lung fields consistent with a diagnosis of hyaline membrane disease. PAP was found as the predominant pulmonary phenotype of the first identified infants with SP-B deficiency, which was not the case for all infants with this genetic disorder. Recently, a study of neonates and children with severe unexplained respiratory distress and PAP revealed that the majority of cases with PAP was not linked to SP-B gene mutations [48].

Histologically, SP-B deficiency in humans has been associated with PAS-positive eosinophilic material in the alveoli, epithelial cell desquamation, enlarged alveolar macrophages with lamellar inclusions and abundant accumulation of SP-A [50,50]. Since SP-B was found to be essential for the proteolytic processing of proSP-C [34,51], newborns with hereditarySP-B deficiency show aberrantly processed SP-C in theintra-alveolar lumen [50,52-54]. Furthermore, an abnormal surfactant activity and altered levels of phosphatidylglycerol were observed in SP-B deficient patients [42,52,55].

Although SP-B deficient neonates usually present with respiratory failure in the first 24 to 48 hours of life, the clinical diagnosis can sometimes be delayed since affected infants may show initially mild symptoms and do not require ventilation or further medical support for some time. The lung disease however is rapidly progressive and fatal respiratory failure finally sets in within 3–6 months, even with maximal medical efforts [56] and lung transplantation is suggested as the only effective treatment [57]. Despite a possible, transient beneficial effect, exogenous surfactant in SP-B deficient infants was found to be largely ineffective [58,59]. This lack of response of surfactant replacement in SP-B deficient infants may be due to an additional function of SP-B precursors, to the accumulation of aberrant SP-C which may inhibit surface film formation or due to an impairment of SP-B recycling [10].

#### Partial surfactant protein B deficiency

There is increasing evidence that some SP-B mutations may result in milder pulmonary phenotypes. In mice with heterozygous disrupted SP-B production, the reduced SP-B synthesis led to chronic lung damage when exposed to hyperoxia [60]. Exogenous SP-B corrected this oxygen-induced pulmonary dysfunction [61].

So far, four patients with partial SP-B deficiency are described in the literature. Characteristics of these patients are described in table 1 (Additional file 1).

##### Case 1 (mutation: G135S)

A male neonate presented during the first hours of life with respiratory distress and required mechanical ventilation. The patient developed persistent pulmonary hypertension and was treated with continous oxygen therapy. Biochemical analyses of his tracheal aspirate fluid revealed a complete absence of SP-B and an aberrant form of SP-C at 12 kd, suggesting the diagnosis of hereditary SP-B deficiency. A genetic analysis demonstrated a point mutation in exon 5 on one SP-B allele and the mother was found to be the carrier. Histological sections of the lung revealed PAP. The authors suggested that this patient had a transient SP-B deficiency caused by a SP-B gene variant. The patient developed chronic lung disease and was alive at 3 years of age requiring oxygen supplementation [62]

##### Case 2 (mutation: 121ins2/R236C)

The most severe course of partial SP-B deficiency was a full-term infant who developed tachypnea shortly after birth. A chest radiogram showed bilateral pneumonitis. Because of poor oxygenation, the infant received extracorporeal membrane oxygenation (ECMO) and remained oxygen dependent for 9 months. The patient showed a beneficial response to corticosteroids and withdrawals of steroid therapy worsened his status dramatically. At 8 months of age, no SP-B was detected in BAL and a genetic analysis revealed a heterozygous state for the 121ins2 mutation. The other allele carried a point mutation (R236C). The mother was identified as the carrier for the 121ins2 and the father of the R236C allele. Since his pulmonary situation deteriorated rapidly, he was accepted for lung transplantation but died at the age of 9 months of unexplained sudden respiratory failure and cardiac arrest.

##### Cases 3 & 4 (mutation: c.479G>T)

Two unrelated children homozygous for a mutation in exon 5 (479G→T) of the SP-B gene have been reported. The genetic defect resulted in reduced SP-B levels and an unclear lung disease. The first patient was born at 40 weeks of gestation and showed RDS at 8 hours of age. Furthermore, he developed a spontaneouspneumothorax at 18 hours of age. He required continous oxygen supplementation and dexamethasone seemed to have no influence onhis respiratory symptoms. At 5 weeks of life, he underwent an open lungbiopsy. Hisrespiratory function declined progressively and he underwentbilateral lung transplantation at 4 months of age.

The second patient was a full-term female who developed RDS shortly after birth. The patient did not require mechanical ventilation but had a persistentoxygen requirement. At 2 months ofage, an open lung biopsy was performed which showed interstitialfibrosis and PAP. Diuresis, steroid therapy and cyclophosphamide therapy had no beneficial effect on her pulmonary symptoms and she developed pulmonary hypertension andright ventricular hypertrophy. At 6 months ofage she was discharged with home oxygen therapy and has been stable for several years with persistent oxygen requirement [63].

The histological phenotype of partial SP-B deficiency included accumulationof extracellular proteins, atypical macrophages, epithelial-celldysplasia and pulmonary fibrosis. However it remained unclear whether these pathologicalfindings were caused by impaired surface tension, by accumulationof abnormal SP-B or by other factors that may causethese forms ofILD. The patients with partial SP-B deficiency were bearing uncommon mutations leading either to the production of small amounts of SP-B or to proSP-B which was not further processed to mature SP-B [62-64]. A critical SP-B level required for physiological lung function has been suggested, which is supported by experiments in mice which developed lung disease when SP-B levels were genetically downregulated below 20–25% of physiological values [65]. Since SP-B levels in the reported cases were not evaluated during the course of disease, the association between levels of SP-B and respiratory status could not be assessed.

#### Surfactant protein B polymorphisms

Polymorphisms in the SP-B gene or an interaction between SP-A and SP-B gene products may act as genetic determinants for the susceptibility to RDS [66-68]. The SP-B gene contains two polymorphisms, the Ile131Thr locus and a polymorphic site in intron 4. A length variation in intron 4 has been proposed to associate withRDS independently [69] and in combination with an SP-A allele [70]. The Ile131Thr polymorphism affects a putative N-linked glycosylation site of proSP-B which may play a role in the genetic susceptibility to RDS in premature neonates. Furthermore, the SP-B Ile131Thr polymorphism may be linked to an unknown genetic element that increases the risk of RDS in cooperation with SP-A allelic variants [66,68]. A large study of premature neonates found no association of the two SP-B polymorphisms with RDS, but showed that the association between SP-A alleles and RDS was dependenton the SP-B Ile131Thr genotype [71]. A consanguineous pedigree with 14 infants dying of neonatal RDS had biochemically a SP-B deficiency. Molecular analyses showed nine SP-B polymorphisms but none of them could explain the observed SP-B deficiency. Interestingly, in lung tissue of these patients aberrant SP-B mRNA was found [72]. A further study of SP-A and SP-B genetic variants in singletons and twins found no direct genetic influence on the risk of RDS [73].

### Surfactant protein C

#### Structure and biosynthesis

SP-C is the only surfactant-associated protein that isexclusively expressed in typeII pneumocytes [74]. The SP-C gene is encoded on the short arm of human chromosome 8 and contains 6 exons. The mature SP-C is encoded in exon 2 and the sixth exon is being untranslated. SP-C is synthesizedby proteolytical processing of the larger precursor protein proSP-C [54]. The SP-C gene is transcribed into an 900 bp mRNA and is translated into the 197 amino acid proprotein (proSP-C). Alternative splicing atexon 5 results in a shorter transcript of 191-amino acid proprotein but the physiological role of the splice variant is not known [75]. The SP-C precursor protein isrouted together with the SP-B precursor protein to multivesicularbodies, where both proteins are processed and packaged into lamellarbodies for secretion into the alveolus [51]. ProSP-C is extensively processed posttranslationally, similar to proSP-B. Recently, the proteases cathepsin H [30] and Napsin A [76] were found to be involved in the first N-terminal processing step of proSP-C. Furthermore, the SP-C peptide is modified posttranslationally by palmitoylation at cysteine residues at positions 5 and 6 [54]. The mature SP-C peptide of 35-amino-acids is stored in the lamellar body from where it is secreted into the alveolar space.

#### Surfactant protein C deleted mice

While SP-B was shown to play a critical role in neonatal lung function, SP-C seems to be involved in a different way.

##### Swiss black outbred mice [35]

The initial strain of SP-C knock-out mice (Swiss black outbred) was viable and had normal lung function. Lung tissues showed subtle pulmonary abnormalities with regular lamellar bodies, reduced viscoelasticity and decreased stability of surfactant bubbles. SP-C mRNA was not detected in the lungs of these mice and no mature SP-C protein was found. BAL fluid levels of SP-A, SP-B and SP-D were similar compared to wild-type mice and pulmonary structures were normal with no indication of inflammation. The number of type II pneumocytes was similar compared to wild type and well-organized lamellar bodies and tubular myelin were detected in the SP-C -/- strains. However, lung mechanics showed a lower hysteresivity (mechanical coupling between tissue resistance and elastance) in SP-C -/- mice at the end-expiratory pressure (low PEEP), suggesting that SP-C stabilizes the phospholipid film at reduced lung volumes. The surface activity of large aggregate surfactant from SP-C -/- mice was similar to SP-C wild-type mice, but the stability of small bubbles was decreased in surfactant of SP-C -/- mice.

This SP-C -/- genetic model showed marked differences compared to the SP-B knock-out model and suggests, (1) that SP-C is not essential for the synthesis or secretion of pulmonary surfactant, (2) that SP-C plays an important role in the stabilization of phospholipid packing at small bubble radius and (3) that SP-C is involved in the recruitment of phospholipids from the subphase to the surface film. Thus, SP-C may play a more important role in the stabilizationof surfactant rather than in its synthesis [35].

##### 129/Sv strain mice [77]

Interestingly, when these SP-C-deficient mice were generated in a different genetic background (129/Sv strain) they developed a more severe lung disease. While lung structure was normal at birth, a progressive air space enlargement, emphysema, macrophage infiltration, abnormal tissue lipid accumulation and pulmonary fibrosis was observed within 2 months after birth. At the age of 6 months, parenchymal infiltrates and type II cell hyperplasia were found. Furthermore, these lungs showed activation of metalloproteases, fragmentationof elastin fibers and infiltration of myofibroblasts in thealveolar septa, similar to findings ofILD in humans. The pulmonary alterations increased progressively with age and at one year of age, the mice developed pneumonitis and emphysema. Analysis of lung mechanics revealed that lung volumes were significantly increased in SP-C -/- mice as compared to wild type mice, consistent with the histologically observed emphysema. Biochemical analyses showed normal levels of SP-B while levels of SP-A and SP-D were increased.

This knock-out mouse model shows that lack of SP-C causes chronic lung disease depending on the genetic background suggesting that SP-C associated lung disease may be triggered by environmental factors and/or modifier genes [77].

#### Surfactant protein C deficiency in human lung disease

There is increasing evidence linking SP-C deficiency to various types of ILD in humans [10,78,79]. While SP-B deficiency generally results in fatal neonatal lung disease, abnormal expression of SP-C has been shown to present as a much more variable phenotype [78,80-89]. Amin et al. found an association between abnormalities in SP-C expression and familial ILD. Three family members with chronic ILD had no detectable levels of SP-C and decreased levels of SP-A and SP-B in their BAL fluid. Immunostaining showed a decreased expression of proSP-C in the alveolar epithelial cells, but no mutations in the exons of SP-B or SP-C were found. Thus the underlying mechanism responsible for the lack of SP-C in these patients remained unknown [90].

Table 2 (Additional file 1) summarizes reported cases of ILD associated with SP-C gene variants. Since the clinical courses showed a large variance, each report is described separately.

#### Case report on SP-C deficiency with mutation c.460+1 G>A / Δexon 4 [83]

The first report on SP-C deficiency was a female infant whose family history showed a three-generation history of ILD inherited in an autosomal-dominant way. The mother was given a diagnosis of DIP at one yearof age and she had been treated with glucocorticoids until the age of 15 years. Her father had diedfrom an unclear chronic lung disease without a diagnosis. The women delivered a full-term baby with no respiratory symptoms at birth. This infant developed tachypnea and cyanosis at six weeks of age and chest radiographs showed interstitial involvement. An open-lung biopsy was performed andshowed NSIP. Mature SP-C could not be detected. Sequencing of the SP-C gene revealed a single base donor splice site mutation with substitution of the first base of intron 4 (c.460+ 1G > A) leading to the skipping of exon 4 and a shortened SP-C proprotein. This mutation was identified on only one allele of the SP-C gene suggesting an autosomal-dominant way of inheritance. The patient's mother also had reduced expression of proSP-C and the same SP-C mutation was found. The girlwas treated with oxygen and glucocorticosteroids andher respiratory symptoms improved. After delivery the lung disease of the mother worsened and she died from respiratoryfailure.

Furthermore, Nogee et al. analyzed the SP-C gene sequence in 34 infants with chronic lungdisease of unknown etiologyand found heterozygous mutations in 11 of 34 patients. Similar to the initially described patient, these mutations led to skippingof exon 4, expression of an aberrant proSP-C and decreased expression of normal SP-C. In 10 infants, with 6 showing a family history of ILD, missense SP-C mutations resulting in amino-acid substitutions (P30L, I73T, G100V, Y104H, P115L, I126R, T187N and L188R)and a frameshift mutation (140delA) associated with expressionof a stable transcript, were identified. Recently, neonates with severe RDS carrying mutations in the SP-C gene were described [91]. Exon 4 deleted SP-C constructs concentrated in perinuclear aggregates which have been shown to be toxic for the developing mouse lung [92]. Furthermore, co-transfection with wild-type and exon 4 deleted SP-C constructs resulted in a pattern similar to the mutant SP-C construct, suggesting a dominant-negative mechanism in heterozygous SP-C mutations [93].

#### Familial SP-C deficiency with mutation c.588 T>A / L188Q [81]

Thomas reported a large kindred of 16 individuals with adults suffering from UIP and children from cellular NSIP. Genetic analyses found a heterozygous T>A transversion at exon 5 + 128 which led to a substitution of a glutamine for leucine at the highly conserved amino acid position 188 of the C-terminus of proSP-C, a region which is essential for intracellular protein trafficking. Immunostaining showed an aberrant subcellular localization of SP-C precursor protein, dense fibrosis and distorted cellular architecture with alveolar type II cell atypia and abnormal lamellar bodies. These findings were similar to those which have been described in type II cellular death or apoptosis [94,95]. When mouse type II cells (MLE cells) were transfected with this SP-C mutation, a similar morphology was found with atypical inclusions resembling lamellarbodies. Furthermore, expression of the mutant protein resulted ina threefold increase of cytotoxicity compared to the wild-type.

Since the identical SP-C mutation caused different pathologic phenotypes (UIP, NSIP) in affected relatives, it has been suggested that SP-C dependent lung diseases may represent pleiotropic manifestations of the same underlying pathogenesis and the mutation may result indifferent histological patterns dependent on the age of manifestation. It is also possible that in this family, cellular NSIP in the affected children occurred as a precursor lesion to UIP in adulthood, but the discrimination of theseentities is discussed controversial [96-98]. Interestingly, two members of the index family with heterozygous SP-C mutations were unaffected. Therefore, the mutation in this kindred showed incomplete penetrance and secondary genetic and/or environmental modifiers may affectthe disease progress.

#### Case report on SP-C deficiency with mutation in exon 3 [86]

A full-term female of two parents without a history of pulmonary disease thrived normally until the age of 3 months. Then, she developed respiratory symptoms with diffuse interstitial infiltrates. The airway surfactant contained decreased levels of mature SP-B and SP-C. An open lung biopsy at 6 months of age revealed an interstitial pneumonitis without an underlying cause. Minimum pulmonary surface tension was increased. SP-C mRNA was present in normal size and amount, but proSP-C was aggregated within type II cells in a compartment separate from SP-B. The cells contained both normal and disorganized lamellar bodies. A genetic analysis was performed and revealed a 9-bp deletion in exon 3 of the SP-C gene. This in-frame mutation spanning codons 91–93 was present on one allele only. Since the parents did not carry this deletion a *de novo *mutation was suggested. The patient's lung function and physical capacity declined dramatically and a bilateral lung transplantation was performed with 14-months of age. The patient's course ameliorated and at 30 months she was breathing ambient air and was gaining weight.

#### Case reports on SP-C deficiency with mutation c.243 T>C / I73T and c.525 G>A / R176Q [80]

In a group of 34 sporadic and familial cases with RDS, in which SP-B mutations had been excluded, two distinct heterozygous SP-C missense mutations were found. The first case was a full-term boy with a normal neonatal course who developed slight respiratory symptoms at the age of 1 month. At age of 9 months, he failed to thrive and his dyspnoea worsened countinously, so he required oxygen supplementation. A subsequent lung biopsy showed PAP combined with ILD. PAP has not yet been described before in patients with SP-C gene variants. Treatment with repetitive whole-lung lavage and systemic corticosteroids and azathioprine resulted in an amelioration of his pulmonary symptoms. Since biochemical BAL fluid analysis showed normal levels of SP-B and SP-C but aberrant forms of proSP-C, a genetic analysis of the SP-C gene was performed. A missense mutation (c.243 T > C) in exon 3 of the SP-C gene was identified, which resulted in the substitution of threonine for a highly conserved isoleucine (I73T). This mutation was not detected in the parents. Similar to the other SP-C mutations, this mutation was heterozygous, consistent with dominant inheritance [88]. In contrast to the initial patient of Nogee [83], where no SP-C could be detected, in this patient mature SP-C protein was present together with an abnormally processed proSP-C suggesting that the mutation altered the processing of proSP-C but allowed the production of mature SP-C.

Brasch et al [88] characterizedthe intracellular trafficking of the I73T mutation *in vitro *using a EGFP/proSP-C^I73T ^fusion protein. When transfected into A549 cells, the mutant proSP-C was routed to early endosomes which is in contrast to previously described SP-C mutations which led to the formation of intracellular proSP-C aggregates [80,93].

Recently, a further *de novo *I73T mutation was found in a full-term infant with PAP and ILD showing a more severe clinical course as compared to the previous case with this mutation. After recurrent bacterial infections, the patient died from respiratory failure at the age of 19 months [99].

The second SP-C mutation of Tredano et al. was found in two patients with PAP from the endogamous white settler population of Réunion Island in which unexplained respiratory distress (URD) has an unexpectedly high prevalence. These two patients had a similar age at the onset of disease of 9 months. However, one infant died from lung disease at age of 18 months, while the other one showed a mild ILD, which was treated by repetitive therapeutic BALs. Both patients showed an abnormal proSP-C, and genetic analysis of the SP-C gene revealed a 2125G-A transition in exon 5, leading to an arginine 167 to glutamine change (R167Q) in the C-terminus of the precursor protein, which is crucial for proper folding and trafficking of proSP-C [93].

#### Case report on SP-C deficiency with mutation g.1509 G>A / E66K [82]

A full-term male showed no post-natal respiratory problems until day 13 when he was admitted to a local hospital with tachypnea, cyanosis and hypoxemia. Chest X-ray showed diffuse bilateral pulmonary infiltrates and he required mild ventilatory support. A lung biopsy showed marked thickening of alveolar septa, mild interstitial chronic inflammation and hyperplasia of type II pneumocytes. The histological diagnosis of PAP and NSIP was esthablished. Immunostaining yielded detectable levels of SP-A, SP-B, SP-C and SP-D in BAL fluid. A DNA sequence analysis found a heterozygous mutation in exon 2 of the SP-C gene (1509 G>A) leading to substitution of a lysine for glutamic acid at codon 66 (E66K). Histologically, proSP-C expression was detected in small endosome-like vesicles. Further *in vitro *trafficking studies were performed using the EGFP/hSP-C^E66K ^fusion protein and showed, that the proSP-C^E66K ^protein accumulated in early endosomes in contrast to Δexon4 and L188Q mutant constructs which formed intracellular aggregates.

#### Surfactant protein C polymorphisms

Two single nucleotide polymorphisms (SNPs) in the SP-C coding sequence resulting in 4 allelic variants have been described. One is localized in codon 138 that encodes threonine or asparagines and the other one in codon 186 that codes for serine or asparagine. The allelic frequencies or the effect of these SNPs on proSP-C processing or function are not known. Several other SNPs in non-coding regions of the SP-C gene have been reported, but also their functional relevance remained unclear [100-102]. Lahti et al. analyzed the association between exonic SP-C gene polymorphisms and their susceptibility to RDS [103]. The study showed that SP-C polymorphisms were associated with RDS and with premature birth but the strength of association varied depending on to the gender of the infants. Recently, Lawson et al found 10 SNPs in the SP-C gene sequence of 13 of 135 analyzed adults with sporadic UIP and NSIP but not in controls. Thus, the majority of sporadic cases of ILD in adulthood may not be associated with SP-Cgene variants [104].

#### Immunomodulatory functions of surfactant protein C

**Figure 1:**
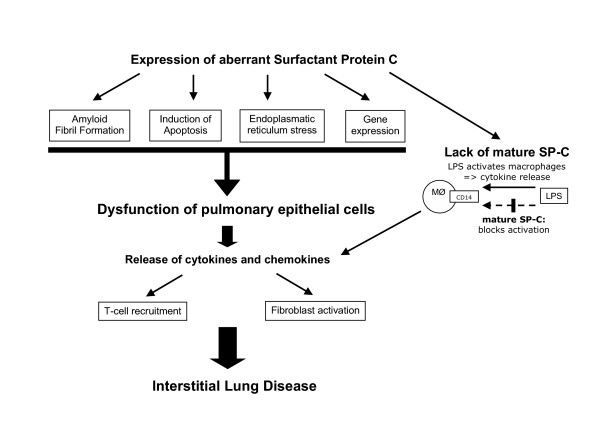
Pathways in the pathogenesis of surfactant protein-C associated interstitiallung disease (according to Beers 2004 [74]). LPS: lipopolysaccharide; MØ: macrophage

While the collectins SP-A and SP-D are well known to participate in innate immune response, a possible role of SP-C in pulmonary immunity has been elucidated only recently. Inhalationof bacterial lipopolysaccharide (LPS) induces acute airway inflammation [105] and long-term exposure to LPS increases the risk of developing chronic lung disease [106]. It has been shown, that the hydrophobic regions of SP-C bind Lipid A, a portion of LPS, in a specific and competitive way. Furthermore, SP-C was found to interact with CD14, the cellular LPS pattern recognition receptor which plays a central role in bacterial-induced inflammation. The SP-C/CD14 interaction blocked the binding of LPS to CD14 and inhibited the effect of LPS on macrophages (Figure 1). This resulted in a decrease of pro-inflammatory cytokine (TNF-α) and NO release by alveolar and peritoneal macrophages in response to LPS [107]. However, an *in vitro *study showed that SP-C didnot influence the direct binding of LPS to CD14, suggesting that other receptors on macrophages, e.g. toll-like receptor 4 or the complement receptor CD11/CD18 may be more essential in this mechanism [108].

These findings indicate thatSP-C plays a role in innatelung defense by preventing LPS-induced pulmonary inflammation. SP-C may either neutralize inhaledLPS or may act as a shuttle to allow the binding of LPS to distinct receptors on alveolarmacrophages. These immunological findings may explain why exogeneous SP-C showed a beneficial effect in a porcine model of LPS-induced lung injury [109].

#### Pathogenesis of surfactant protein C associated lung disease

The current model of SP-C associated lung disease suggests two mechanisms how aberrant forms of SP-C may cause interstitial lung disease.

• Heterozygous mutations in the SP-C gene produce an aberrant proSP-C protein. This aberrant protein interacts with the regular intracellular pathway of SP-C biosynthesis. Consequently, this leads to inhibition of functionally active SP-C production and results in a SP-C deficiency in the alveolus.

• When aberrant proSP-C is produced in large amounts, it cannot be handled by the protein degradation pathway and induces cellular injury and pulmonary inflammation resulting in interstitial lung disease. Figure 1 depicts several hypothesized pathways in the pathogenesis of SP-C associated ILD according to Beers [78].

SP-C associated lung disease may be caused either by a lack of mature SP-C, by the accumulation of aberrant, toxic proSP-C or by both mechanisms. In humans with SP-C mutations, additional factors, e.g. viral infections, hypoxia or drugs, may trigger the accumulation of misfolded proprotein as the SP-C clearance pathway may operate at its limiting capacity. Since SP-C is suggested to be involved in thealveolar reuptake of surfactant particles and plays a role in innate immunity, it remains unclear which specific activity causes the lung diseasein patients with SP-C mutations.

#### BRICHOS domain

Recently, a conserved domain of ~100 amino acids, termed BRICHOS domain, has been identified in several previously unrelated proteins. These proteins include the BRI family of proteins (linked to familial British and Danish dementia), chondromodulin-I associated with chondrosarcoma, CA11 protein associated with stomach cancer and the distal region of proSP-C (F94-I197) associated with ILD. In most of these proteins, the BRICHOS domain is localized in the propeptide region that is removed during proteolytic processing [110]. Mutations in the SP-C gene can be classified depending on the involvement of the BRICHOS domain (Table 2, Additional file 1, and more detailed in [78]). *In vitro *trafficking experiments showed that constructs of BRICHOS domain mutations, e.g. hSP-C^Δexon4 ^[83] and hSP-C^L188Q ^[81], form large toxic intracellular aggregates, termed aggresomes [82,93]. In contrast, mutations outside the BRICHOS domain, as hSP-C^E66K ^and hSP-C^I73T^, led to aberrant proSP-C accumulation in a constitutive endosomal pathway which may inhibit the cellular surfactant recycling system [78,82,88]. Clinically, mutations in the BRICHOS domain resulted in a more severe pulmonary phenotype, mostly associated with death in the neonatal period, while non-BRICHOS mutations led to a milder clinical course.

#### Amyloid fibril formation

Amyloid fibril formation represents a potential mechanism how misfolded higher-order structures of SP-C proteins may cause epithelial cell damage. At least 20 incurable diseases are associated with amyloid fibrils including Alzheimer's disease and spongiform encephalopathy [111]. The metastable α-helical domain of SP-C was found to transform under certain circumstances into energetical favored β-sheet aggregates, which polymerize to an amyloid-like fibril formation [112]. Such amyloid-like fibrils were isolatedfrom the BAL fluids of a PAP patient and in low amounts of three healthy controls [113]. The pathophysiological relevance of these findings remains unclear but suggest that conditions, which alter the secondary structure of SP-C in terms of predisposing to intracellular fibril formation, may have deleterious effects on type II cells. These findings support the model of a conformational lung disease due to a misfolded SP-Cprotein [78]. Destabilizing point mutations may enhance the transition of regular SP-C structure to pathological forms [78].

### ATP-Binding cassette (ABC) transporters

#### ABCA3

The ATP-binding cassette (ABC) transporters aremembrane proteins that utilize the energy of ATP hydrolysis to translocate solutes across cellular membranes. Fourteen ABC genes have been associated withdistinct genetic diseases in humans including Tangier-disease and cystic fibrosis [114,115]. ABCA3 is a 1704 amino acid proteinwhichis highly expressed in type II epithelial cells at the limiting membraneof lamellar bodies. The structureand localization of the ABCA3 transporter suggest a potentialrole in intracellular lipid homeostasis of lamellar bodies [116]. Mutations in the ABCA3 gene are the most recently described inborn error of surfactant metabolism [16]. Shulenin et al. identified mutations in the ABCA3 as a cause of fatal RDS in newborn infants and theunderlying cause of chronic ILD in one child living up to 6 years of age. In 21 infantswith severe neonatal surfactant deficiency in which SP-B and SP-C mutations had been excluded, the coding regions of the ABCA3 genewere sequenced. Furthermore, lung tissue from four patients was analyzed. In 16 of the 21 patients, nonsense, frameshift and splice sites mutations were found. These patients included five consanguineous families. The consanguinity and a family historyof neonatal respiratory distress suggest that the lung disease associatedwith mutations in the ABCA3 gene were inherited in an autosomal-recessive manner. A mutation on one allele was identified in two patients who showed a similarclinical presentation as compared to the remaining patients. Therefore the authors speculate that these children may have a second mutation within introns or in regulatory regions which was not detected by sequencing the coding regions. The radiological findings and clinical symptoms of infants with ABCA3 mutations were similar to newborns with SP-B deficiency. One infant with a missense mutation on one allele was still alive at six years of age showing lung histology of a ILD/DIP, suggesting that some ABCA3 mutations are not fatal and may result in a milder course of disease. Histological examination in these patients showed type II cells hyperplasia and accumulation of alveolar macrophageswith proteinaceous materialand interstitial thickening, indicating DIP and neonatal PAP. Electron microscopy revealed abnormally small lamellar bodies indicating that the ABCA3 transporter is critical for proper formation of the lamellar bodies. A defectivetransport of lamellar-body associated lipid components may result in abnormal processing and structure of surfactant. Furthermore, the mutant ABCA3 transporter may transfer lipids which are deleteriousto the function of alveolar surfactant. These findings suggest that a high percentage of unclear casesof lethal neonatal lung disease may be due to mutations in the ABCA3 gene and affected families could benefit from genetic counseling.

Since there are no therapies known for the lung disease due to ABCA3 mutations, the majority of affected newborns die from respiratory failure in the neonatal period. Except for one patient, the phenotype of non-fatal lung disease caused by ABCA3 mutations is unknown and requires screening for ABCA3 mutations in neonates and children with severe ILD where no underlying cause can be found [16].

#### ABCA1

A recent *in vitro *study showed that surfactant synthesis and export are associated with a basolateral lipid efflux pathway which is mediated by ABCA1, suggesting a role for ABCA1 in the regulation of cellular content and composition of pulmonary surfactant [117]. Targeted disruption of the ABCA1 locus resulted in distinctive pulmonary features [118]. Macroscopically, multiple pale foci were found in the lungs which increased in severity withage, affecting <10% of the parenchyma in 7 months-old miceto 30% in 18 months-old mice. These lesionsconsisted of foamy hypertrophic type II cells, alveolar macrophages and cholesterol clefts. Furhermore, alveolar septae were thickened by aggregates of lymphocytes and plasma cells. In more severe lesions, pulmonary architecture collapsed due to hypertrophic and hyperplastic type II pneumocytes. Oil red staining showed lipid accumulation within alveolar cells and electron microscopy identified these cells as type II pneumocytes and intraalveolar macrophages. The possible role of ABCA1 in ILD is unknown and there are no reports on ABCA1 mutations associated with lung disease in humans.

### Genes involved in lung morphogenesis, surfactant protein expression and lamellar body synthesis

Besides SP-B, SP-C and ABCA3, there is a number of genes involved in lung morphogenesis, surfactant expression and lamellar body function which may also play a role in ILD in children.

Lung morphogenesis is a complex process mediated by numerous transcription factors [11,119], growth factors [120,121] and other signalling molecules that coordinatealveolar cell proliferation and differentiation. Factors playing important rolesin lung morphogenesis include thyroid transcription factor-1(TTF-1) [122-124], GATA-6 [125,126], Foxa2 [127], Hoxb5 [128], TGF-β via SMADs [129,130], CREB [131], Glucocorticoid Receptor [132], HNF-3β [133], N-myc [134] and members of the Gli family [119]. Knock-out mice of these genes have provided insight into the function during lung development [119,135]. For example, Foxa2 expressionwas found to be restricted to subsets of respiratory epithelial cells and was co-expressed with TTF-1, CCSP, SP-A, SP-B, SP-C and SP-D [127,136]. In a mouse model, deletion of Foxa2 in pulmonary epithelialcells inhibited the differentiationof respiratory epithelial cells and resulted in neonatal respiratoryfailure with hyaline membranes as typically found in preterm infants with RDS.

#### TTF-1

TTF-1 is a member of the Nkx2.1 family which is involved in thyroid and lung epithelial-specific gene expression [124,137,138]. Co-transfection assays showed that TTF-1 activity is stimulatedby HNF-3β and GATA-6 which are co-expressed with TTF-1 in the developing lung [139]. While TTF-1 serves overall as an important regulator of surfactant protein gene expression [140], a regulatory role for HNF-3β has been reported only for SP-B [141] and CCSP [142] expression. Furthermore, TTF-1 and HNF-3β were shown to functionally interact in the regulation of the SP-B and CCSP promoter activity [122,143]. TTF-1 deficient pulmonaryepithelial cells failed to express mature SP-B, SP-C and CCSP [124,144]. Deletion of TTF-1 in mice caused thyroid and lungabnormalities with tracheal-esophageal fistulaand dysgenesis of the peripheral lung resulting in respiratoryfailure at birth [145,146]. In humans, mutations in the TTF-1 gene were associated with chorea, hypothyroidism and respiratory distress in newborns [147-149]. Furterhmore TTF-1 gene variants were found in older children with choreoathetosis, hypothyroidism and respiratory distress [150].

#### Growth factors and cytokines

Various growth factors and cytokines have been described to regulate surfactant protein gene expression in the fetal lung [121]. Epidermal growth factor (EGF) was found to increase SP-C gene expression [151], while TGF-β and TNF-α inhibited surfactant protein expression. In mice homozygous for a targeted deletion of the EGF receptor, defects in surfactant function and in SP-A and SP-C expression were observed [152]. The inhibitory effect of TGF-β on surfactant protein gene expression in the fetal lung was found to be mediated at the transcriptional level [26] through SMAD3 interactions with TTF-1 and HNF-3β [153].

#### Lamellar body associated molecules

Lamellar bodies have an acidic interior (pH≈5.5) and contain lysosomal enzymes (e.g. cathepsins C and H) and lamellar body associated membrane proteins (LAMP; e.g. LAMP1 [154], LAMP2 [155] and LAMP3 [156]). Recently, the dendritic cell-lysosomal associated membrane protein (DC-LAMP) was detected intracellularly and at the cell surface of type II pneumocytes. Since type II cells constitutively express MHC class II molecules, it is suggested that they may act as antigen-presentingcells. Since DC-LAMP is expressed on type II cells and on dendritic cells, a relationship between DC-LAMP and MHC II function in type II pneumocytes has been suggested [157,158]. Recently, a study showed that the proteins t-SNARE, syntaxin 2 and SNAP-23 were expressed in alveolar type II cells and were associated with the plasma and lamellar body membrane. SNAP23 was found to be required for the fusion of lamellar bodies with the plasma membrane. An antisense oligonucleotide for syntaxin 2 inhibited surfactant secretion. These results suggest that syntaxin 2 and SNAP-23 are required for regulated surfactant exocytosis in type II cells [159].

The characterization of molecules involvedin surfactant protein gene expression, lung morphogenesis and lamellar body synthesis expands the current model how a network of genes may cause, trigger or promote lung disease. However, most of these genes causedlethal lung malformations rather than chronic lung diseases and were only studied in animal models [11]. Nevertheless, the potential role of these genes in pediatric lung disease is still unclear and requires further investigation. Insights on how these molecules are involved in human lung physiology may reveal new candidate genes for identifying mutations associated with ILD in children.

#### Hermansky-Pudlak syndrome

In the autosomal-recessive disorder Hermansky-Pudlak-Syndrome (HPS), most prevalent among Puerto Ricans, the biogenesis of lysosomes and lysosome-related organelles is affected. In humans, six different forms of HPS have been described referring to the genes HPS 1 to 6. In patients with HPS, the molecular defect leads to hypopigmentation, bleeding disorders and fibrotic lung disease, mostly UIP. The incidence and severity of pulmonary involvement in HPS patients varies widely but the forms HPS1 and HPS4 were most likely associated with ILD [160-162]. Lung tissue from HPS patients with ILD exhibits giant lamellar bodies in type II epithelial cells, patchy fibrosis and airspace enlargement [163].

## Conclusion

### Surfactant protein B

The lethal lung disease in SP-B deficient mice and humans demonstrates the crucial role for SP-B in neonatal lung function. The accumulation of aberrant proSP-C in SP-B deficiency strongly suggests an important intracellular role for SP-B in the processing of SP-C. The clinical phenotype of hereditary SP-B deficiency presents typically as full-term neonates with severe respiratory distress syndrome refractory to standard therapies, includingventilation and surfactant replacement. Lung transplantation is regarded as the only effective treatment. Chest X-rays are similar to those of premature infants with respiratory distress syndrome showing diffuse ground glass pattern. Alveolar proteinosis is the predominant pathological phenotype. The SP-B 121ins2 mutation is suggested to account for up to two-thirds of the mutant alleles identified in the SP-B gene. The overall incidence of hereditary SP-B deficiency can be estimated from the allele frequency of the 121ins2 mutation of 1 per 1000–3000 individuals. Of great interest for clinical practice are some cases with partial SP-B deficiency. These patients showed less severe clinical phenotypes and prolonged survival into childhood with chronic lung disease and oxygen dependency as compared to fully SP-B deficient patients. Thus, it is likely that partial SP-B deficiency may be the underlying cause in some children with unexplained chronic lung disease.

### Surfactant protein C

SP-C mutations were found to be associated with ILD in neonates and children with sporadic or autosomal-dominant inheritance. The majority of these mutations were missenses or deletions leading to skipping of exon 4 or substitution of highly conserved amino acids in the region of the proSP-C molecule. *In vitro *studies support the hypothesis that the identified SP-C mutations were causally related to the associated lung disease. The phenotype covers a wide range of clinical features and age of onset. Reported symptoms include respiratory distress with dyspnoea, tachypnea, cyanosis, asthmatic bronchitis and failure to thrive. The histological diagnosis was mostly NSIP, in some cases combined with PAP. Adults with SP-C mutations showed UIP or DIP while other subjects remained asymptomatic. The phenotype of lung disease associated with SP-C mutations may represent pleiotropic manifestations of the same underlying pathogenesis. Affected individuals may carry a genetic risk for interstitial lung disease, which becomes apparent in dependence of the genetic background, i.e. modifier genes and environmental influences. These findings have essential implicationsfor the diagnosis of ILD in children as genetic diagnoses can be made and underlying etiologies of familiar cases of ILD may be identified. In children with ILD, where no underlying cause can be found, the patient's DNA, BAL and lung tissue and the parents' DNA should be conserved for genetical, biochemical and histological analysis. Since the incidence and prevalence of ILD associated with SP-C gene mutations are unknown it is necessary to analyse larger subsets of clinically well defined patient cohorts.

### ABCA3

Mutations in the ATP-binding cassette transporter ABCA3 gene are the most recently described hereditary disorders of surfactant metabolism. In 16 of 21 patients, who had no mutations in the SP-B or SP-C gene, mutations in ABCA3 were found inherited in an autosomal-recessive manner. Clinical symptoms and radiological findings of infants with ABCA3 mutations were similar to newborns with SP-B deficiency. Histological diagnoses included DIP, neonatal PAP and CPI. Interestingly, one infant with a missense mutation on one allele was still alive at six years of age showing lung histology of DIP, suggesting that ABCA3 mutations may also result in a milder course of disease. These findings indicate that a high percentage of unclear casesof lethal neonatal lung disease may be due to mutations in the ABCA3 gene and affected families could benefit from genetic counseling. Except for one patient, the phenotype of non-fatal lung disease caused by ABCA3 mutations is unknown and requires screening for ABCA3 mutations in neonates and children with severe ILD where no underlying cause can be found.

Table 3 (Additional file 1) gives an overview of pediatric ILD associated with surfactant protein deficiency or ABCA3 mutations. In full-term neonates with unexplained, severe respiratory distress, refractory to therapeutical efforts, genetic analysis of the SP-B and ABCA3 coding sequences are warranted. In children with progressive interstitial lung disease, where no underlying cause can be found, a genetic analysis of the SP-C gene may be useful, especially in cases with a family history of unclear lung disease.

## Abbreviations

ABCA1 ATP-binding cassette transporter A1

ABCA3 ATP-binding cassette transporter A3

CPI chronic pneumonitis of infancy

DIP desquamative interstitial pneumonitis

GM-CSF granulocyte-macrophagecolony-stimulating factor

ILD interstitial lung disease

LAMP lamellar body associated membrane protein

LPS lipopolysaccharide

NSIP non-specific interstitial pneumonitis

PAP pulmonary alveolar proteinosis

RDS respiratory distress syndrome

SNP single nucleotide polymorphism

SP surfactant protein

TTF-1 thyroid transcription factor-1

URD unexplained respiratory distress

UIP usual interstitial pneumonitis

## Competing interests

The author(s) declare that they have no competing interests.

## Authors' contributions

DH and MG wrote the manuscript and read and approved the final version.

## Supplementary Material

Additional File 1 **Table 1**. Pathological features and clinical phenotypes of reported cases with partial SP-B deficiency. **Table 2. **Pathological features and clinical phenotypes of reported cases with mutations in the SP-C gene. **Table 3. **Overview: Pediatric interstitial lung disease associated with surfactant protein deficiency or ABCA3 mutations Click here for file
