# Supplementary material for: Interstitial lung disease in children – genetic background and associated phenotypes
Source: Respir Res. 2005 Apr 8;6(1):32. doi: 10.1186/1465-9921-6-32 (PMC1090616; doi:10.1186/1465-9921-6-32)
Supplement: Additional File 1 — Table 1. Pathological features and clinical phenotypes of reported cases with partial SP-B deficiency. Table 2. Pathological features and clinical phenotypes of reported cases with mutations in the SP-C gene. Table 3. Overview: Pediatric interstitial lung disease associated with surfactant protein deficiency or ABCA3 mutations [file 1465-9921-6-32-S1.doc]

# Interstitial lung disease in children – genetic background and associated phenotypes

### Dominik Hartl and Matthias Griese

## Tables

### Table 1. Pathological features and clinical phenotypes of reported cases with partial SP-B deficiency

**Tables**

| **Mutation** | **Histology** | **Immunohistochemistry** | **Surface tension** | **Radiological signs** | **Pulmonary Symptoms** | **Family carrier** | **Family history** | **Initial symtoms** | **Clinical Course** | **Reference** |
| --- | --- | --- | --- | --- | --- | --- | --- | --- | --- | --- |
| c.417G>A  G135S | PAP   - Proteinaceous material - Inflammatory cells - Intraluminal tubular myelin | - SP-B: - - Aberrant proSP-C (12kd) - SP-A: + | Not done | Not reported | - Pulmonary hypertension - Pulmonary hemorrhage | No carriers | - Negative - Not related | First hour of life: chylothorax | - Mechanical ventilation - Continous oxygen therapy - 3 years of age: oxygen supplementation - Now: chronic lung disease | Klein et al.[1] |
| 121ins2/  R236C (heterozygot)  exons 4 & 7 affected | - Interstitial fibrosis - Type II hyperplasia - Proteinaceous   material - Findings similar but   less marked than SP-  B deficiency | - SP-B in BAL: -   Tissue: + (low amount)   - Aberrant proSP-C (6, 18kd) - SP-A + - proSP-B (25, 43 kb) | 25 mN/m (controls<10) | Bilateral Pneumonitis | Pulmonary hypertension | *Paternal:* R236C  *Maternal:*  121ins2 | Maternal grandfather died at 41 years of asthma | Shortly after birth  Dusky skin and increasing tachypnoea | - Hospitalized the whole life - Remained oxygen dependent - Died at 9.5 months of age of a sudden respiratory and cardiac arrest | Ballard et al.[2] |
| g.2479G>T  c.479G>T  exons 5 & 7 affected | - Eosinophilic material in the alveolar space - Foamy macrophages - Desquamated pneumocytes - Noncompact irregular lamellar bodies - Type II cell hyperplasia | - Aberrant proSP-C (~12kd) - SP-B – - ProSP-B - | Not done | RDS | RDS | Not done | - Negative - Not related | - Respiratory distress at 8 hours of life   => mechanical  ventilation   - Spontaneus pneumothorax at 18h of age | - Respiratory distress symptoms deteriorated   => bilateral lung transplantation at 4 mo of age | Dunbar et al.[3] |
| g.2479G>T  c.479G>T  exons 5 & 7 affected | Interstitial fibrosis & PAP   - Type II cell hyperplasia - Eosinophilic material in the alveolar space - Foamy macrophages - Desquamated pneumocytes - Noncompact irregular   lamellar bodies | - Aberrant proSP-C (~12kd) - SP-B + (variable, 12-16%) - ProSP-B + | Not done | RDS | - RDS - Pulmonary hypertension | Not done | - Negative - Not related | Respiratory distress shortly after birth | - Persistent oxygen requirement, - Discharge at home at 6 mo of age with home oxygen therapy - Progressive course with pulmonary hypertension and right ventricular hypertrophy - Now 6 years of age, stable for several years with persistent oxygen requirement | Dunbar et al.[3] |

PAP: pulmonary alveolar proteinosis; RDS: respiratory distress syndrome, SP-B -: no SP-B detectable, SP-B +: SP-B detectable

Table 2. Pathological features and clinical phenotypes of reported cases with mutations in the SP-C gene

| **Mutation** | Histology | **Immunohistochemistry** | **Radiological signs** | **Pulmonary Symptoms** | **Family carrier** | **Family history** | **Initial symtoms** | **Clinical Course** | **References** |
| --- | --- | --- | --- | --- | --- | --- | --- | --- | --- |
| g.1727G>A  c.460+1 G>A  Δexon4 | NSIP   - Well-preserved pulmonary   architecture,  hyperplasia of type II alveolar cells  interstitial infiltrate of  lymphocytes with scattered myofibroblasts.   - Normal lamellar bodies | - Aberrant proSP-C + (weak) (~ 20 kd) - SP-B + - ProSP-B + - SP-C - | Increased interstitial markings | - Dyspnoea - Tachypnea - Respiratory insufficiency | Mother | Mother: DIP (diagnosed at 1 year of age) | 6 weeks of age:  tachypnea and cyanosis | Treated with supplemental  oxygen and corticosteroids => respiratory symptoms improved | Nogee et al.[4] |
| c.460+1 G>T Missense  Δexon4  (P30 L, I73T,  G100V, Y104H, P115 L, I126R, T187N, and L188R  Frameshift (140delA)  P30 L  “non-BRICHOS domain”  mutation | - Similar to c.460+1 G>A   Pneumonitis (unspecified) | Similar to c.460+1 G>A | Similar to c.460+1 G>A | Similar to c.460+1 G>A | No carriers | - 11 patients with SP-C mutations - 6 with a family history of lung disease | Not reported | Not reported  continued... | Nogee et al.[5] |
| g.2188T>A,  c. 588T>A  L188Q | *children:*  NSIP  *adults:*  DIP/UIP   - Large, dysplastic, cuboidal type II cells - Fibrocystic pulmonary dysplasia | Aberrant proSP-C + | *Infants:*  Ground glass appearance with fine fibrillary infiltration  *Adults:*   - Bilateral interstitial fibrosis with infiltrates, worse at bases - Extensive, scattered nodular infiltrations - Diffuse coarse reticulation with multiple lucencies - Reticulonodular   interstitial infiltrates   - Bilateral patchy interstitial opacities and honeycombing | *Infants:*   - Respiratory failure - Failure to thrive   *Adults:*   - Dyspnea - cough - clubbing | Positive  heterozygous | Kindred:   - 11 adults and - 3 children with pulmonary fibrosis. | Not reported | *Adults:*   - 4 alive - 7 death of pulmonary fibrosis   *Children:*  1 death with 1 year of age at respiratory insufficiency | Thomas et al.[6] |
| Δ91–93  ΔExon 3  9bp deletion  “non-BRICHOS domain”  mutation | - Interstitial pneumonitis - Hyperplasia of type II pneumocytes - Alveolar septal widening with a mild lympho-plasmacytic infiltrate - Cholesterol clefts | - SP-A ++ (increased) - SP-C – (mRNA +) - ProSP-C + - SP-B + (decreased) | Diffuse interstitial infiltrates | - Dyspnoea, - Respiratory insufficiency | No carriers | - Negative - Not related | healthy until 3 mo of age, then   - Growth failure - Difficulty   feeding, - Dyspnoea | - Progressive decline in pulmonary function - Bilateral lung transplantation at 14 mo of age. - At 30 mo of age, breathing ambient air, gaining weight | Hamvas et al.[7] |
| g.2125G>A  c.525G>A  R167Q | PAP | - SP-C - Aberrant ProSP-C +  (11, 13 kd) | Alveolar and interstitial  infiltrates, lung distension,  mediastinal lymph nodes | - Dyspnoea, - Tachypnea - Anemia - Failure to thrive | No carriers | - Negative - Not related | With 9 months  Respiratory distress | - One child: died at 18 months of intractable hypoxemia, - Other child:  alive with moderate ILD   continued... | Tredano et al.[8] |
| g.1286T>C  c.243 T>C  I73T  “non-BRICHOS domain”  mutation | PAP and NSIP   - PAS-positive material - Thickening of alveolar septa - Hyperplasia of type II pneumocytes - Abnormal vesicular organelles | - SP-A + - ProSP-B + - SP-B + - SP-C + - Aberrant ProSP-C +  (11, 13 kd) | Diffuse bilateral  Pulmonary infiltrates | - Dyspnoea, - Tachypnea - Failure to thrive - Clubbing | No carriers | - Negative - Not related | Slight dyspnoeaa  and tachypnoea at the age of 1 month.  highly | - At 1 mo: dyspnoea, tachypnoe - 3 months: recurrent bronchitis - Progressive dyspnoea - Failure to thrive - Alive with persistent O2 supplementation and high dose glucocrticosteroids and azathioprine | Brasch et al.[9] |
| g.1286T>C  c.243 T>C  I73T  “non-BRICHOS domain”  mutation | PAP and NSIP   - Widened alveoli with thickened alveolar septa - Fibroblasts and lymphocytes infiltration - Hyperplasia of type II pneumocytes | Not done | Diffuse ILD | - Dyspnoea, - Tachypnea | No carriers | - Negative - Not related | At 3 mo episodes of asthmatic bronchitis | - 5 mo : delay in motorneuro development - 9 mo: progressive failure to thrive - Recurrent respiratory infections - Progressive need for O2 supplementation - Acute respiratory failure - Mechanical ventilation, - Died at respiratory insufficiency | Percopo et al.[10] |
| g.1509 G>A  E66K  “non-BRICHOS domain”  mutation | PAP and NSIP   - Marked thickening of alveolar septa due to spindle-shaped cells, - Mild interstitial chronic inflammation, - Hyperplasia of type II pneumocytes | - SP-A + - SP-B + - SP-C + - SP-D + - ProSP-C +  (90% Type II cells)   Increased surface tension  (20 mN/m, normal <5) | Diffuse bilateral  pulmonary infiltrates | - Dyspnoea, - Tachypnea - Respiratory insufficiency | No carriers | - Negative - Not related | No immediate perinatal problems  13th day:   - Tachypnea, - Cyanosis - Hypoxemia | Mild ventilatory support | Stevens et al.[11] |

DIP: desquamative interstitial pneumonitis; ILD: Interstitial lung disease; NSIP: non-specific interstitial pneumonitis; PAP: pulmonary alveolar proteinosis;
RDS: respiratory distress syndrome; UIP: usual interstitial pneumonitis, SP-B -: no SP-B detectable, SP-B +: SP-B detectable

**Table 3. Overview: Pediatric interstitial lung disease associated with surfactant protein deficiency or ABCA3 mutations**

|  | **Hereditary SP-B deficiency** | **Partial SP-B deficiency** | **SP-C deficiency** | **ABCA3 mutation** |
| --- | --- | --- | --- | --- |
| Age of onset | Neonatal | Neonatal | Variable | Mostly neonatal or within 3 months after birth,  one case with longer survival (6 years of age) |
| **Typical symptoms** | Surfactant deficiency,  Respiratory failure | Variable  Respiratory failure Pulmonary hypertension | Variable  Tachypnea,  Cyanosis | Surfactant deficiency, Respiratory failure |
| Inheritance | Recessive | Recessive | Dominant or sporadic | Recessive |
| **Cause** | Absent SP-B | Decreased SP-B | Abnormal ProSP-C | Unknown |
|  |  |  | Reduced mature SP-C |  |
| Pathology | - abundant alveolar concentric multilamellated structures and membranous vesicles - alveoli filled with eosinophilic material (positive for periodic acid–Schiff) - abundant accumulation of SP-A and proSP-C - enlarged alveolar macrophages with lamellar inclusions, dense granules, and myeloid bodies | - accumulation of extracellular proteins - atypical alveolar macrophages - epithelial-cell dysplasia - interstitial fibrosis | - interstitial fibrosis - hyperplasia of alveolar type II cells - accumulation of alveolar macrophages with various amounts of proteinaceous material | - hyperplasia of alveolar type II cells, - accumulation of alveolar macrophages with various amounts of proteinaceous material, - interstitial thickening, - smaller lamellar bodies with dense inclusion bodies |
| **Diagnosis** | Congenital alveolar proteinosis, RDS | RDS, Pulmonary fibrosis | Alveolar proteinosis NSIP, DIP, UIP | Alveolar proteinosis,  DIP, CPI |

CPI: chronic pneumonitis of infancy; DIP: desquamative interstitial pneumonitis; NSIP: non-specific interstitial pneumonitis; RDS: respiratory distress syndrome; UIP: usual interstitial pneumonitis

References

1. Klein JM, Thompson MW, Snyder JM, George TN, Whitsett JA, Bell EF *et al*.: **Transient surfactant protein B deficiency in a term infant with severe respiratory failure.** *Journal of Pediatrics* 1998, **132:** 244-248.

2. Ballard PL, Nogee LM, Beers MF, Ballard RA, Planer BC, Polk L *et al*.: **Partial Deficiency of Surfactant Protein-B in An Infant with Chronic Lung-Disease.** *Pediatrics* 1995, **96:** 1046-1052.

3. Dunbar AE, Wert SE, Ikegami M, Whitsett JA, Hamvas A, White FV *et al*.: **Prolonged survival in hereditary surfactant protein B (SP-B) deficiency associated with a novel splicing mutation.** *Pediatric Research* 2000, **48:** 275-282.

4. Nogee LM, Dunbar AE, Wert SE, Askin F, Hamvas A, Whitsett JA: **A mutation in the surfactant protein C gene associated with familial interstitial lung disease.** *New England Journal of Medicine* 2001, **344:** 573-579.

5. Nogee LM, Dunbar AE, Wert S, Askin F, Hamvas A, Whitsett JA: **Mutations in the surfactant protein C gene associated with interstitial lung disease.** *Chest* 2002, **121:** 20S-21S.

6. Thomas AQ, Lane K, Phillips J, Prince M, Markin C, Speer M *et al*.: **Heterozygosity for a surfactant protein C gene mutation associated with usual interstitial pneumonitis and cellular nonspecific interstitial pneumonitis in one kindred.** *American Journal of Respiratory and Critical Care Medicine* 2002, **165:** 1322-1328.

7. Hamvas A, Nogee LM, White FV, Schuler P, Hackett BP, Huddleston CB *et al*.: **Progressive lung disease and surfactant dysfunction with a deletion in surfactant protein C gene.** *American Journal of Respiratory Cell and Molecular Biology* 2004, **30:** 771-776.

8. Tredano M, Griese M, Brasch F, Schumacher S, de Blic J, Marque S *et al*.: **Mutation of SFTPC in infantile pulmonary alveolar proteinosis with or without fibrosing lung disease.** *American Journal of Medical Genetics Part A* 2004, **126A:** 18-26.

9. Brasch F, Griese M, Tredano M, Johnen G, Ochs M, Rieger C *et al*.: **Interstitial lung disease in a baby with a de novo mutation in the SFTPC gene.** *European Respiratory Journal* 2004, **24:** 30-39.

10. Percopo S, Cameron HS, Nogee LM, Pettinato G, Montella S, Santamaria F: **Variable phenotype associated with SP-C gene mutations: fatal case with the I73T mutation.** *European Respiratory Journal* 2004, **24:** 1072-1073.

11. Stevens PA, Pettenazzo A, Brasch F, Mulugeta S, Baritussio A, Ochs M *et al*.: **Nonspecific Interstitial Pneumonia, Alveolar Proteinosis, and Abnormal Proprotein Trafficking Resulting from a Spontaneous Mutation in the Surfactant Protein C Gene.** *Pediatric Research* 2004, **19**.
